# Genome‐resolved metagenomics revealed novel microbial taxa with ancient metabolism from macroscopic microbial mat structures inhabiting anoxic deep reefs of a Maldivian Blue Hole

**DOI:** 10.1111/1758-2229.13315

**Published:** 2024-09-12

**Authors:** Lapo Doni, Annalisa Azzola, Caterina Oliveri, Emanuele Bosi, Manon Auguste, Carla Morri, Carlo Nike Bianchi, Monica Montefalcone, Luigi Vezzulli

**Affiliations:** ^1^ Department of Earth, Environmental and Life Sciences (DiSTAV) University of Genoa Genoa Italy; ^2^ National Biodiversity Future Center (NBFC) Palermo Italy; ^3^ Department of Integrative Marine Ecology (EMI) Stazione Zoologica Anton Dohrn—National Institute of Marine Biology, Ecology and Biotechnology, Genoa Marine Centre (GMC) Genoa Italy

## Abstract

Blue holes are vertical water‐filled openings in carbonate rock that exhibit complex morphology, ecology, and water chemistry. In this study, macroscopic microbial mat structures found in complete anoxic conditions in the Faanu Mudugau Blue Hole (Maldives) were studied by metagenomic methods. Such communities have likely been evolutionary isolated from the surrounding marine environment for more than 10,000 years since the Blue Hole formation during the last Ice Age. A total of 48 high‐quality metagenome‐assembled genomes (MAGs) were recovered, predominantly composed of the phyla *Chloroflexota*, *Proteobacteria* and *Desulfobacterota*. None of these MAGs have been classified to species level (<95% *ANI*), suggesting the discovery of several new microbial taxa. In particular, MAGs belonging to novel bacterial genera within the order *Dehalococcoidales* accounted for 20% of the macroscopic mat community. Genome‐resolved metabolic analysis of this dominant microbial fraction revealed a mixotrophic lifestyle based on energy conservation via fermentation, hydrogen metabolism and anaerobic CO_2_ fixation through the Wood–Ljungdahl pathway. Interestingly, these bacteria showed a high proportion of ancestral genes in their genomes providing intriguing perspectives on mechanisms driving microbial evolution in this peculiar environment. Overall, our results provide new knowledge for understanding microbial life under extreme conditions in blue hole environments.

## Graphical abstract

This study investigates macroscopic microbial mat structures found in complete anoxic conditions at 70 m depth in a Maldivian Blue Hole. Genome‐resolved metagenomic led to the description of several new taxa with ancient metabolism revealing novel bacterial genera belonging to the order Dehalococcoidales as dominant members of these communities.

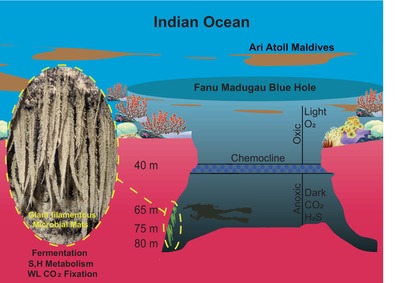

## INTRODUCTION

Blue holes are subsurface cavities that form in carbonate banks through processes of dissolution and/or fracture‐type collapse of rocks, under the influence of sea level changes (Gischler, 2011). These environments mostly originated during the last glacial period, when sea level was 100–120 m below current levels and exposed the bedrock to weathering thus becoming submerged by the subsequent rise in sea level (Smith et al., 2011). Blue Holes were filled with water during the past approx. 15,000 years (Fairbanks, 1989) and can be marine, fresh, or of mixed chemistry (Mylroie et al., 1995). The relatively enclosed geometry of the cavities results in little or no water exchange, so the internal environment is usually stable; limited water exchange fosters the vertical stratification of water column geochemistry and the sharp zonation of biological communities, thus creating unique environments that can be defined extreme from an ecological point of view (Gischler et al., 2013; van Hengstum et al., 2020).

Inland blue holes are commonly characterized by a surface freshwater layer overlying saline groundwater, with a transition zone marked by a halocline (Björnerås et al., 2020). Oceanic blue holes, which form along coastlines and in coral reefs (Wyrwoll et al., 2006), do not have freshwater layers, all the water they contain being fully marine. However, water column stability creates vertical gradients of temperature, dissolved oxygen, and other chemical factors. Consequently, a chemocline forms to separate water layers with different redox/chemical processes, leading to anoxic and sulphidic bottom waters where aerobic organisms are replaced by facultative and strict anaerobic microbial communities normally involved in active sulphate reduction processes (Canganella et al., 2007; van Vliet et al., 2021). These autotrophic and/or heterotrophic prokaryotes, which may form large biofilms on rocky surfaces or on bottom sediments, are especially adapted to anoxic/microaerophilic conditions and are typically distinct from those of any other marine and freshwater systems (Patin et al., 2021).

Currently, the best studied examples of oceanic blue holes come from the western Atlantic and the South China Sea (Bottrell et al., 1991; Gischler et al., 2008; Sun et al., 2023). However, knowledge on true marine blue holes is still limited if compared with their inland counterpart. Several blue hole studies focussed on their geological and hydrological aspects (Kovacs et al., 2013; Whitaker & Smart, 1997; Xie et al., 2019) but these environments have also been identified as natural laboratories that have the potential to foster evolutionary studies and to investigate phenotypic diversity, adaptation and speciation of resident organisms (Langerhans, 2017).

The Faanu Mudugau Blue Hole (FMBH), located at 3°55.507′N, 72°56.559′ E in the Ari Atoll, is the first discovered in the Maldives (Betzler et al., 2015; Bianchi et al., 2009) and one of the few described for the Indian Ocean (Wyrwoll et al., 2006). Faanu Mudugau is a peripheral reef on the eastern rim of Ari Atoll: the blue hole opens at about 30 m depth, at the bottom of a large bowl shaped depression and has a roughly circular entrance measuring 70 m in diameter. Its walls overhang an irregular bottom covered by fine carbonate loose sediments at 70–85 m depth. Hydrogen sulphide is found in water deeper than 45–50 m (Colantoni et al., 2003; Cutroneo et al., 2023). Between 65 and 75 m depth, macroscopic microbial mat structures are found attached to the steep sidewalls, reaching up to 15 cm in length. Microbial mats are generally found in extreme habitats where they are not outcompeted or grazed by other organisms (Kindler et al., 2022; Paerl et al., 2000) and comprise stratified microbial communities that vary taxonomically and functionally depending on the environment in which they form (Wong et al., 2020). In FMBH such communities have likely been physically and chemically isolated from the surrounding marine environment since the blue hole formation, thus offering a unique opportunity to investigate the biology of mat‐building microbes with a distinct evolutionary history and adaptation strategies in one of the most extreme habitats found in the oceans. Although vertical stratification of microbial communities in the water column has been previously investigated in blue hole environments (Chen et al., 2023; Patin et al., 2021), little is known about sessile microbial formations permanently found below the chemocline in deep blue hole layers which are likely to represent one of the most successful examples of life adaptation to such harsh and isolated environment.

In this study, macroscopic microbial mat structures found in complete anoxic conditions at 70 m depth in FMBH were sampled in 2022, and their biology investigated by employing a genome‐centric metagenomic approach.

## EXPERIMENTAL PROCEDURES

### Study site

**FIGURE 1:**
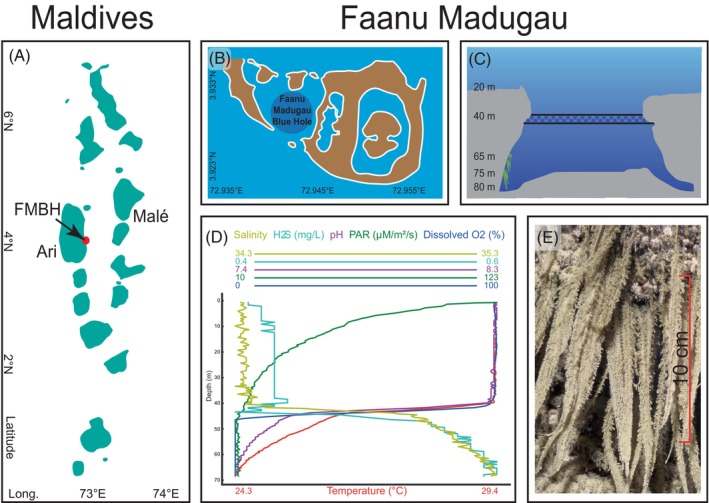
Study site description: (A) Map of the Ari Atoll in the Maldivian Archipelago. (B) Faanu Mudugau lagoon situated on the eastern side of the Ari Atoll, showing the position of the FMBH. (C) Schematic cross‐section illustrating the structure of the FMBH, location of the chemocline is shown around 45–50 m depth, microbial mats location is indicated green. (D) Chemical and physical profiling conducted along the water column of the FMBH (modified from Cutroneo et al., 2023). (E) macroscopic microbial mat structures sampled in this study.

The Archipelago of the Maldives is composed of 26 atolls in the central Indian Ocean, stretching for 800 km in a north–south direction from about 7°06′N to 0°45′S in latitude and 72°33′E to 73°45′E in longitude (Godfrey, 2018). The shape of the Maldivian atolls is that of ‘leaking buckets’ (Betzler et al., 2015), originated according to the antecedent karst model, which envisages their formation as resulting from karst dissolution that occurred through exposure during glacial lowstands of sea level (Droxler & Jorry, 2021; Purdy & Bertram, 1993) and deglacial reflooding when sea level rose again in the Late Quaternary (Rovere et al., 2018; Rufin‐Soler et al., 2014). Multifarious evidences of antecedent karst corrosion are recognizable in the coral reefs of the Maldives, such as high dolomite content in the sea floor, patch reefs, and submerged notches and caves (Bianchi et al., 1997). Among the latter, the Blue Hole of Fanu Mudugau is an outstanding example (Figure 1A–C). First discovered in 1999 by the diver Massimo Sandrini, it was explored and mapped between 2000 and 2003 (Colantoni et al., 2003). FMBH is a hypogenic cavity the roof of which collapsed during the last Ice Age (Würm stage, 115.000–11.700 years ago). Finding speleothemes, such as stalactites and stalagmites, at about 50 m depth provided the ultimate proof of the Quaternary emersion of the Maldivian archipelago and of its karst origin (Bianchi et al., 2009). In the bell‐shaped cavity three depth‐related zones can be distinguished by their physico‐chemical and biological characteristics (Azzola et al., 2022; Cutroneo et al., 2023; Doni et al., 2024) (Figure 1D). (1) From the entrance (30 m depth) to about 40 m, waters are oxygenated, chlorophyll‐rich, with higher pH, temperature, and conductivity values. Aerobic microbes dominate and the walls are covered by a variety of macrobenthic autotrophic and heterotrophic species; fish penetrate only near the entrance. (2) Around 45–50 m depth, a thick chemocline separates the upper oxic layer and the deep anoxic layer. In this transitional zone temperature, conductivity, pH and Eh dramatically decrease. Macroscopic life is absent but patches of microalgae cover the rock. Both aerobic and anaerobic (obligate or facultative) bacteria are found, causing a peak in total microbial metabolism and thus suggesting that the oxic‐anoxic interface represents a highly metabolically active zone. (3) Below 50 m oxygen (O_2_) concentration rapidly collapses, while both carbon dioxide (CO_2_) and hydrogen sulphide (H_2_S) greatly increase. In this anoxic and dark zone, with low pH and Eh, there are no macroorganisms but a few meiofaunal nematodes can survive (Sandulli et al., 2006, 2014) and macroscopic microbial mat structures are found (Figure 1E and Video S1).

### Sampling

During the 25th Maldives Scientific Cruise organized by the University of Genoa and Albatros Top Boat in May 2022, macroscopic microbial mat structures were manually sampled in triplicate from the sidewall of the FMBH at approximately 70 m depth by diving scientists (Figure 1E). For sample collection, sterile 50 ml conical Falcon tubes were used. Mat samples were then preserved with RNA Later and stored at room temperature. In addition, water samples (8 L) were also collected along the water column at 33, 43, and 64 m depth using 10 L sterile tanks. On board, water samples were immediately filtered onto self‐preserving 0.45 μm PES filters using an eDNA automatic sampler (Smith‐Root Inc., Vancouver) and stored at room temperature.

### Microscopic analysis of microbial mat samples

A portion (1 g) of the microbial mat samples were immediately fixed in 4% paraformaldehyde. The samples were studied using different microscopical analysis. (i) Light microscopy: samples were spread on a microscope slide, let to dry and stained with a dye mixture (Stain Solution I containing Eosin Y; Stain Solution II containing Thiazine Dye) (Giemsa, Diff‐Quik, Medion Grifols Diagnostics AG, Switzerland) and observed under a light microscope (Olympus IX53 microscope, Milano, Italy). (ii) Fluorescent microscopy: samples were stained with an Acridine Orange solution for 5 min then washed and observed by fluorescent microscopy (Exc = 490–500 nm; Em = 520 nm) (Thunder Imager 3D cell culture of Leica Microsystems). Field emission scanning electron microscopy (FESEM): samples were filtered by vacuum pump on Whatman 0.2 μm polycarbonate filter, dehydrated in an ascending series of ethanol washes (25%, 40%, 60%, 80%, 90%, and 100%, each step 10 min), and air‐dried. Then samples were sputter‐coated with graphene and viewed using a UHR FE‐SEM Tescan Clara, HV, 5 keV, 300 pA, in‐beam axial detector.

### Nucleic acid extraction

Upon arrival in the laboratory, the microbial mat samples (1 g) were pooled and washed twice and DNA extraction was carried out using the Qiagen DNeasy PowerSoil (QIAGEN srl, Milan, Italy) following the manufacturer's instructions. DNA from seawater filters was extracted using the PowerWater Pro Kit (QIAGEN srl, Milan, Italy) following the manufacturer's instructions. DNA concentration was estimated by the QuantiFluorTM dsDNA System using a QuantiFluorTM fluorometer (Promega Italia srl, Milano, Italy). Sizing of genomic DNA was also conducted in an Agilent Bioanalyzer 2100 (Agilent, Palo Alto, CA) using the High Sensitivity DNA kit (Agilent Technologies).

### Analysis of seawater samples by 16S rRNA gene‐based profiling of the microbial community

Metagenomic DNA extracted from three water samples collected at different depth (33, 43, and 64 m corresponding to oxic, chemocline and anoxic waters, respectively) in the FMBH was amplified using primers targeting the V4‐V5 hypervariable region of the 16S rRNA gene of bacteria using the primer set 515F‐Y (5′‐GTGYCAGCMGCCGCGGTAA‐3′) and 926R 5′‐CCGYCAATTYMTTTRAGTTT‐3′ described by Parada et al. (2016). Amplicons libraries were prepared according to the Illumina “16S Metagenomic Sequencing Library preparation” protocol (15044223 Rev. B) and run on an Illumina MiSeq platform (2×300bp) to obtain at least 200 K reads per sample. A total of 789,458 paired‐end reads spanning the V4–V5 hypervariable region of the bacterial 16S rRNA gene were produced in the analysis. Sequence data were processed using DADA2 (Callahan et al., 2016) in QIIME 2‐2022.8 (Bolyen et al., 2019). Briefly, after the denoising step, including primer removal, quality filtering and chimera removals, Amplicon Single Variants (ASV) were inferred. Following denoising 570,708 trimmed read sequences were obtained, corresponding on average to 95,118 sequence reads per analysed sample and a total of 3947 ASVs were recovered. Taxonomic classification was performed using the SILVA 138 database (Quast et al., 2013) and ASVs affiliated with mitochondria and chloroplast sequences were removed. The ASV abundance data were normalized using the Cumulative Sum Scaling method (Paulson, 2014).

### Metagenomic sequencing of macroscopic microbial mat structures in the FMBH, MAGs reconstruction and taxonomic classification

Genomic DNA extracted from a single pool of three replicate Microbial Mat samples was used to produce an indexed library for next‐generation sequencing on the Illumina platform (Illumina, Inc.) using the KAPA HyperPlus Kit for Illumina (Roche Diagnostics, Mannheim, Germany). Shotgun metagenomic libraries were sequenced by STAB VIDA, LDA company (Caparica, Portugal) on an Illumina HiSeq X Ten platform (2×150bp) producing ca. 20Gbases of sequence read data. Raw shotgun metagenomic sequencing reads were trimmed using the read_qc module from metaWRAP (v 1.1) (Uritskiy et al., 2018). The cleaned reads were assembled with MEGAHIT (v 1.1.2) (Li et al., 2015). Binning was performed using MetaBAT2 (Kang et al., 2019), MaxBin2 (Wu et al., 2016) and Concoct (Alneberg et al., 2014), which was subsequently refined using the bin_refinement module from metaWRAP. The quant_bins module was employed to estimate the abundance of each bin; abundances were expressed as “genome copies per million reads.” Taxonomic inference for each MAG was conducted using GTDB‐tk (v 2.1.0) (Chaumeil et al., 2020), while quality assessment was performed with CheckM (v 1.2.2) (Parks et al., 2015). All sequence reads data were deposited at NCBI Sequence Read Archive, with Accession Number: PRJNA1074233.

### Metabolic analysis of MAGs

Metabolic and biogeochemical functional potential of MAGs were predicted using METABOLIC (v 4.0) (Zhou et al., 2022). METABOLIC integrates annotation of proteins using KEGG, TIGRfam, Pfam, custom hidden Markov model databases, dbCAN2, and MEROPS; incorporates a protein motif validation step to accurately identify proteins based on prior biochemical validation and determines the presence or absence of metabolic pathways based on KEGG modules. The metabolic capabilities at the community scale were determined using the ‘MW‐score’ (metabolic weight score) metrics.

### Phylogenomic analysis of dehalococcoidales MAGs

For extensive phylogenomic analysis of dominant MAGs in the FMBH (*n* = 6), classified at the order level as *Dehalococcoidales* within the phylum *Chloroflexota*, all available *Dehalococcoidales* genomes (*n* = 179) were retrieved from the NCBI database. To construct a robust phylogenomic tree, redundant and low‐quality genomes were removed by de‐replicating at a 99% average nucleotide identity (ANI) threshold using dRep (v 1.4.3) (Olm et al., 2017), with all default parameters except for ‘—contamination 10’ to exclude highly contaminated genomes. This curation yielded 56 *Dehalococcoidales* genomes. These genomes, along with the six MAGs, were then used for ANI and Amino Acid Identity (AAI) calculations. The phylogenomic tree was constructed with GToTree (v 1.8.3) (Lee, 2019) using a set of 74 bacterial single‐copy genes and IQtree (Nguyen et al., 2015). ANI calculations followed the methodology of Richter & Rosselló‐Móra, 2009, as implemented in the pyani Python module (v 0.2.12) (Pritchard et al., 2016). AAI between genomes was calculated with CompareM (v 0.1.2) (Available at: https://github.com/dparks1134/CompareM).

### Identification of ancestral genes in Dehalococcoidales MAGs

To investigate the presence of ancestral genes in *Dehalococcoidales* MAGs, 296 unique COGs identifiers out of 355 protein families potentially present in the Last Universal Common Ancestor (LUCA) were recovered from Weiss et al. (2016). COGs annotation was performed on Dehalococcoidales MAGs and on 44 type strain genomes (Table S1) using Melange (Available at: https://github.com/sandragodinhosilva/melange). The number of LUCA's COGs identified in the MAGs or genomes was then normalized to the total number of annotated COGs for each MAG or genome (LUCA‐likeness, Mushegian, 2008).

## RESULTS AND DISCUSSION

### Microbial community structure in the water column of the FMBH

According to the oxygen vertical profile a shift in the microbial community with increasing depth was apparent in the FMBH. Taxonomic binning and classification of 16S rRNA gene sequences showed that *Alpha*‐, *Gamma*‐*proteobacteria* and *Bacteroidia* were the most abundant in the oxic zone, accounting on average for 16%, 13% and 13% of the total bacterial diversity (Figure S1A). In contrast *Nanoarchea* (8%) and *Omnitropia* (6%) were the most abundant in the deep anoxic layer (Figure S1A). Other anaerobic taxa, such as *Desulfobacterota* and *Dehalococcoidia*, were mostly found in the anoxic layer (Figure S1B). Overall, the composition of microbial communities along the water column in the FMBH is consistent with results from microbial community profiling recently reported in other blue hole environments (Chen et al., 2023).

### Macroscopic microbial mat composition in the FMBH

**FIGURE 2:**
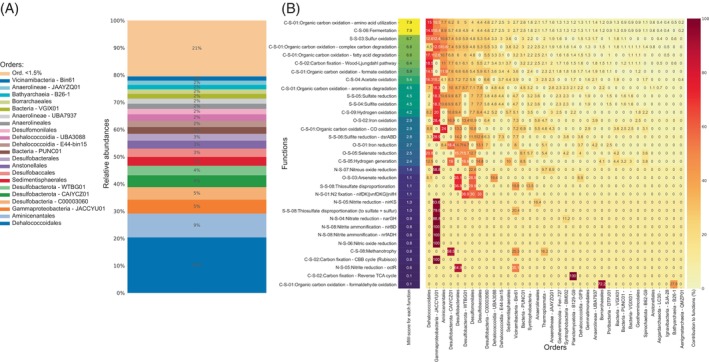
Composition (A) and Metabolic Functions (B) of macroscopic Microbial Mats in the FMBH based on metagenomic data. The left heatmap corresponds to the MW‐score for each function (most abundant functions are listed), and the rest of the table indicates the contribution percentage to each MW‐score of the MAGs grouped at order taxonomic rank. Metabolic Weight Score (MW‐score) is a metric reflecting the functional capacity and abundance of a microbial community in co‐sharing functional networks.

Microscopic examination of microbial mat samples collected in the FMBH revealed the presence of small round‐shaped bacterial cells (~1 μm size) dominating the mat fabric (Figure S2A–C). FESEM analysis of the samples also revealed the presence of abundant pyrite aggregates with framboid texture in the mats (Figure S2D). Shotgun sequencing of mat samples yielded 70,322,373 paired‐end reads from Illumina platforms. *Following the quality control and the trimming*, the de novo assembly produced 1,149,069 contigs with an N50 of 1181 bp. The binning and refinement process of MAGs resulted in the recovery of 48 MAGs, each with a completion ranging from 50% to 99% and contamination <10% (Figure S3). Of these, seven MAGs were classified within the domain *Archaea*, and the remainder were affiliated with *Bacteria*. Quantitative analysis revealed that the most abundant community members were predominantly MAGs taxonomically assigned to the order *Dehalococcoidales*, class *Gammaproteobacteria* and phylum *Desulfobacterota* (Figure 2A). Notably, according to the average nucleotide identity (ANI) values calculated between reconstructed MAGs and the genomes of the closest strains, none of the recovered MAGs could be classified at the species level, suggesting the discovery of several new uncharacterized microbial taxa (Table S2).

### Metabolic functions of microbial mat communities in the FMBH

Metabolic profiling of the microbial mats in FMBH was conducted by calculating MW‐score (metabolic weight score) at the community‐scale level based on results of metabolic profiling and gene coverage from metagenomic read mapping. MW‐score (metabolic weight score) is a metric reflecting the functional capacity and abundance of a microbial community in co‐sharing functional networks. More frequently and higher abundance metabolic functions in a microbial community lead to higher MW‐scores (Zhou et al., 2022).

According to the MW‐scores analysis diverse metabolisms are observed in the mat including a mixotrophic nutritional strategy based on carbon fixation, sulphur metabolisms, fermentation, fatty acid and complex carbon degradation (Figures 2B and S4 and Table S3). Carbon fixation occurred mainly through the Wood–Ljungdahl pathway (WL) in the mat communities of FMBH. This pathway is believed to be one of the most ancient metabolisms for anaerobic carbon fixation (Adam et al., 2018) and still represents an important component of the metabolic machinery in multiple anaerobic prokaryotes (Youssef et al., 2019). In particular, the WL pathway is employed primarily by acetogenic bacteria, archaeal sulphate reducers, or methanogens (Smith et al., 2019) but its presence has also been reported in a wide variety of uncultured anaerobic lineages, including members of the *Dehalococcoidales* (Adam et al., 2018; Zhuang et al., 2014).

Given that inorganic carbon fixation is energetically costly, it was previously suggested that the ability of mixotrophic microorganisms to utilize or switch between different carbon acquisition mechanisms may represent a cost‐effective survival strategy in extreme environments (Hügler & Sievert, 2011). In particular, the WL pathway has been recognized as an additional and highly energy‐efficient method for autotrophic carbon fixation in anoxic zones such as dark anoxic layers of blue hole environments (Chen et al., 2023). Bin_17 belonging to the class *Gammaproteobacteria*, was the only MAG containing genes encoding for the ribulose‐1,5‐bisphosphate carboxylase/oxygenase (RuBisCO) enzyme whilst Bin_34, classified within the phylum *Planctomycetota*, contained genes involved in the reverse Tricarboxylic Acid cycle for carbon fixation. RuBisCO is a key enzyme for carbon fixation through the Calvin–Benson–Bassham (CBB) cycle and is found in plants, algae, cyanobacteria, and many autotrophic bacteria including phototrophs and chemolithotrophs (Southward et al., 1996). Our findings agree with previous studies that identify MAGs possessing the CBB cycle primarily in the class *Gammaproteobacteria* in anoxic waters of blue hole environments (Chen et al., 2023). In the same environments, aerobic phototrophs such as *Synechococcus* and *Alphaproteobacteria* (mainly *Rhodospirillales*) harbouring the RuBisCO enzyme have been mostly reported in upper aerobic layers (Chen et al., 2023).

Sulphur‐metabolism‐related genes were identified in 19 MAGs with the potential to perform elemental sulphur oxidation and/or sulphate reduction. This latter is a dominant respiration pathway in anoxic H_2_S‐rich marine environments (Jørgensen et al., 2019). Accordingly, near half of the MAGs in FMBH had complete pathways for dissimilatory sulphate reduction including genes encoding for ATP sulphurylase (*Sat*), APS reductase (*Apr*) and dissimilatory sulphite reductase (*Dsr*) involved in sulphate reduction to sulphide (Basen et al., 2011). Interestingly, most of the sulphur‐reducing taxa belonged to *Desulfobacterota*. In contrast, the sulphur oxidation pathway was predicted from genes encoding for sulphur dioxygenases (SDOs) that catalyse the oxidation of S^0^ to sulphite whilst the sulphide:quinone oxidoreductase (Sqr) encoding gene, which catalyses the oxidation of sulphide to elemental sulphur, was not detected in the mat community (Sun et al., 2023). Elemental sulphur and thiosulphate are major products of H_2_S oxidation (Yao & Millero, 1993; Zhang & Millero, 1993) and could be formed during the intrusion of oxygenated water into the suboxic/anoxic interface (Ho et al., 2004). However, although SDO can utilizes ferric ion (Fe_3_^+^) as an electron acceptor for elemental sulphur oxidation under anaerobic conditions (Sugio et al., 1987), mechanisms potentially involved in this process in FMBH would be difficult to infer.

Finally, *regarding nitrogen metabolism*, genes involved in N_2_ fixation were identified in Bin_14, Bin_31, and Bin_46, belonging, respectively, to the WTBG01, *Desulfobaccales*, and *Desulfomonilales* orders within the *Desulfobacterota* phylum (referred to as *Deltaproteobacteria* in NCBI taxonomy) suggesting diazotrophic activity in FMBH.

### Phylogenomic and metabolic analysis of dominant Dehalococcoidales taxa in FMBH mat communities

**FIGURE 3:**
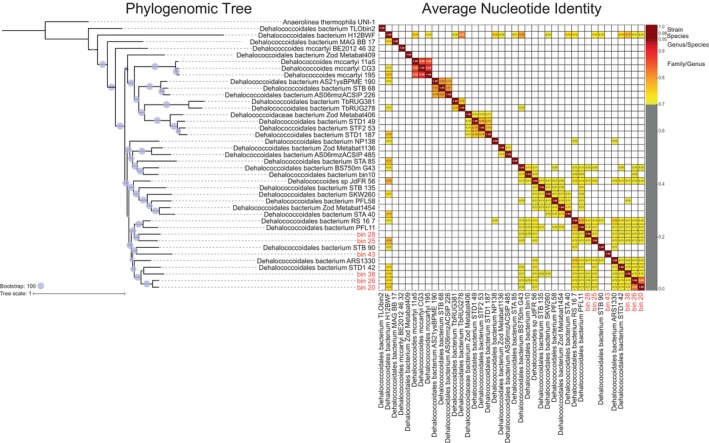
Phylogenomic and Average Nucleotide Identity Analysis of dominant *Dehalococcoidales* taxa in the FMBH mat communities calculated from metagenomic data. *Anaerolina thermophila* was used as the outgroup in the phylogenomic Tree.

MAGs classified at the order level as *Dehalococcoidales* (*n* = 6), belonging to the phylum *Chloroflexota*, represented the most abundant taxa in FMBH accountings for about 20% of the microbial mat community (Figure 2A). The phylogenomic affiliations of the *Dehalococcoidales* MAGs with 56 phylogenetically close reference genomes corroborated their taxonomic classification within the order *Dehalococcoidales* (Figure 3). These MAGs clustered in a distinct branch of the phylogenomic tree (Figure 3), alongside with other unclassified *Dehalococcoidales*. Average Nucleotide Identity (ANI) analysis (Figure 3) showed that *Dehalococcoidales* MAGs did not match with any of the species for which reference genomes are available (genome similarity <95% ANI). In particular, Bin_25, Bin_38 and Bin_43 were related to the *Dehalococcoidales* bacterium H12BWF, with an ANI of 78%, 74% and 69%, respectively. Bin_28 displayed a similarity to *Dehalococcoidales* bacterium RS_16_7, with an ANI of 71%. Finally, Bin_26 and Bin_20 showed a degree of similarity, with an ANI value of approximately 84% and were also related to the *Dehalococcoidales* H12BWF (72% and 71% ANI, respectively). *Dehalococcoidales* H12BWF is a MAG recovered from the bottom layer of the Yellow Sea at a depth of 60 meters (Song et al., 2020). Overall, these ANI values in combination with the completeness of recovered bins (Figure S3) suggest that reconstructed MAGs in the FMBH are distinct from all representative *Dehalococcoidales* genomes available in the NCBI database, precluding a definitive species‐level phylogenetic placement.

To investigate this further, genus‐level placement was assessed using Amino Acid Identity (AAI), where a threshold of 70% is considered as a genus‐level cut‐off (Santos et al., 2021). The results showed an AAI of 84.19% between Bin_20 and Bin_26, confirming their classification within the same genus (UBA5760), in agreement with taxonomic analysis. Furthermore, both Bin_20 and Bin_26 showed an AAI of 69% if compared with Bin_38. However, all MAGs exhibited AAI percentages below the genus‐level cut‐off with reference genomes. Consequently, these findings suggest that the *Dehalococcoidales* MAGs likely represent novel species across different new potential genera within the order *Dehalococcoidales*.

**FIGURE 4:**
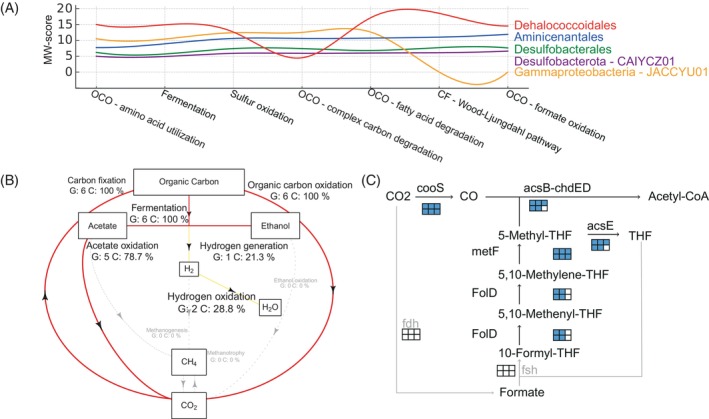
Metabolic analysis of dominant *Dehalococcoidales* MAGs in the FMBH mat communities based on metagenomic data. (A) Identification of the top seven metabolic functions within the mat community. Reconstruction of the (B) Carbon cycle and (C) Wood–Ljungdahl pathway for the six *Dehalococcoidales* MAGs (six‐square boxes). In (B), labels for each arrow indicate: Step number and reaction, number of genomes G capable of conducting these reactions, metagenomic coverage C of genomes (represented as a percentage within the community) that can conduct these reactions. Grey lines indicate the absence of reactions.

According to their high abundance in the mat community, MW‐score analysis identified *Dehalococcoidales* as the most metabolically important taxa underscoring their crucial role in biological functions and processes of macroscopic microbial mats in FMBH (Figure 2). Genome resolved metabolic analysis revealed that MAGs belonging to *Dehalococcoidales* possess a wide range of metabolic capabilities. They were identified as mixotrophs, evidenced by the capability to use both organic carbon sources via fermentation processes and dark carbon fixation pathways, notably acetogenesis via the WL pathway (Figure 4A,B). Interestingly, *Dehalococcoidales* MAGs utilizing the WL pathway were also recently recovered from anoxic waters in the Yongle Blue Hole in the South China Sea (Chen et al., 2023), a geographic distant area which suggests these bacteria could well adapt to selective pressure characterizing such extreme environments.

In acetogenesis, molecular hydrogen (H2) and inorganic carbon (CO2 or HCO3^−^) are metabolized by the WL pathway. This pathway involves the reversible reduction of CO2 into the carbonyl and methyl moieties of acetyl‐CoA, proceeding through two distinct branches: the carbonyl branch and the tetrahydrofolate (THF) methyl branch (Figure 4C). Most *Dehalococcoidales* MAGs in our study exhibited a nearly complete WL pathway, with genes for both the carbonyl and tetrahydrofolate (THF) methyl branches identified (Figure 4C and Table S4). However, they lacked the initial two genes (*fdh* and *fsh*) of the THF methyl branch, which are responsible for converting CO2 to formate and then to formyl‐THF (Figure 4C). Notably, Bin_25 and Bin_28 possessed a formate dehydrogenase linked to coenzyme F420, distinct from the canonical formate dehydrogenases involved in the methyl branch of the WL pathway, which was found in other MAGs across various phyla, including Bin_33, Bin_14, Bin_34, Bin_40, and Bin_42. The formate‐tetrahydrofolate ligase was absent in all *Dehalococcoidales* MAGs, but was present in Bin_10 and Bin_41 (although a complete formate oxidation pathway could not be reconstructed from these sequences), both belonging to the class *Anaerolineae*. A previous study on the incomplete Wood–Ljungdahl pathway in *Dehalococcoides mccartyi* strain 195 showed its capability to incorporate exogenous formate for serine biosynthesis and to cleave acetyl‐CoA, generating methyl‐THF for methionine biosynthesis. This compensates for the lack of *Fdh* and *MetF* functions (Zhuang et al., 2014).

### Evolutionary aspects

*Dehalococcoidales* dominating macroscopic microbial mat community in the anoxic deep layer can be considered one of the most successful organisms in FMBH. Although seawater intrusion from the upper layers could not be excluded, possibly in relation with storms or extreme events such as tsunamis that hit this area in the past (Klostermann et al., 2014; Morri et al., 2015), these bacteria have likely been isolated from the surrounding environment for more than 10,000 years since FMBH formation during the last glacial period. Chemical and physical isolation created opportunities for new species to develop, a statement that is well supported by the detection of novel microbial taxa in our study.

**FIGURE 5:**
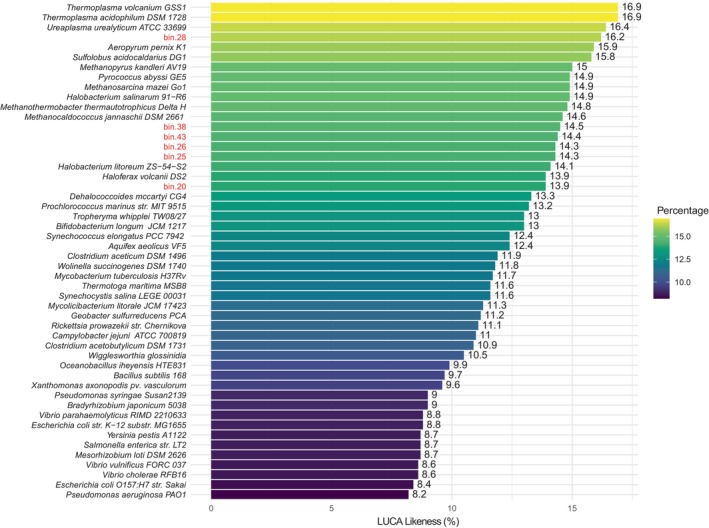
Proportion (%) of last universal common ancestor's clusters of orthologous groups (Weiss et al., 2016) in *Dehalococcoidales* MAGs and reference genomes (LUCA‐likeness, Mushegian, 2008).

The analysis of metabolic pathways and genetic traits in FMBH mat communities, and especially in *Dehalococcoidales* MAGs, showed strong resemblance with traits believed to be present in ancestral life at the dawn of evolution. Accordingly, comparative analysis of COGs in *Dehalococcoidales* MAGs with 296 unique COGs recently proposed to trace to LUCA (Last Universal Common Ancestor) identified 271 shared COGs (Figure 5) (Weiss et al., 2016). Notably, the proportion of ancestral COGs in the Dehalococcoidales MAGs (“LUCA‐likeness”, Mushegian, 2008) was comparable or higher than that of other genomes for microbial species whose modern lifestyles resemble that of LUCA, including *Clostridia* and methanogenic *Archaea* (Figure 5 and Table S5). These results suggest that evolutionary forces linked to extreme conditions found in the FMBH promote the establishment of ancestral traits that were advantageous in such peculiar environment thus providing new insights on mechanisms driving microbial evolution across different time scales.

## CONCLUSION

Genome‐resolved metagenomic analysis of macroscopic microbial mat structures found in permanent anoxic layer in the Maldivian Blue Hole revealed novel microbial taxa whose evolution have most probably been constrained in this environment in the middle of the Indian ocean for thousands of years. Potential new genera within the order *Dehalococcoidales* dominated the mat community and showed a versatile mixotrophic metabolism drove by fermentation of organic substrates and anaerobic CO_2_ fixation through the Wood–Ljungdahl pathway. These bacteria shared several genetic traits with hypothetical organisms that have been recently proposed as ancestral to all life forms providing intriguing perspectives on mechanisms driving microbial evolution in extreme environment across different time scales.

## AUTHOR CONTRIBUTIONS

**Luigi Vezzulli:** Conceptualization; investigation; funding acquisition; writing – original draft; supervision; methodology; validation; project administration. **Lapo Doni:** Conceptualization; investigation; writing – original draft; methodology; validation; software; data curation; formal analysis. **Annalisa Azzola:** Investigation; methodology; validation; writing – review and editing. **Caterina Oliveri:** Investigation; methodology; validation; writing – review and editing; data curation. **Emanuele Bosi:** Investigation; writing – review and editing; validation; methodology; data curation. **Manon Auguste:** Investigation; writing – review and editing; methodology; validation; data curation. **Carla Morri:** Conceptualization; investigation; writing – review and editing. **Carlo Nike Bianchi:** Conceptualization; investigation; writing – review and editing. **Monica Montefalcone:** Conceptualization; investigation; writing – review and editing; supervision.

## CONFLICT OF INTEREST STATEMENT

The authors declare no conflict of interests.

## Supporting information


**FIGURE S1.** Microbial community structure in the water column of the FMBH (A) Relative abundances of Bacterial ASVs in the column water sampled at depths of 33, 43 and 64 m clustered at the class level. (B) Barplot Illustrating the abundance of the *Dehalococcoidia* class in the column water of the FMBH.

**FIGURE S2.** Light (A, B), Epifluorescence (C) and Field emission scanning electron microscopy (FESEM) photographs of microbial mat samples collected in the FMBH. Small round‐shaped bacterial cells (~1 μm size) form a well‐discernible dense network in the mats (a), uncoloured particles (b), filamentous‐like structure (c) and nano‐micron framboidal pyrite aggregates (d) are also visible in the mat fabric.

**FIGURE S3.** MAG statistics and taxonomy. For each MAG, the figure shows quantification, contamination (%), completeness (%), and taxonomy, including phylum, class and order ranks.

**FIGURE S4.** Reconstruction of the carbon, sulphur, and nitrogen biogeochemical cycles at the mat community scale.

**TABLE S1.** LUCA‐Likeness.
**TABLE S2.** MAGs taxonomy and QC.
**TABLE S3.** Metabolic community characterization.
**TABLE S4.** Community WL pathway.
**TABLE S5.** recovered LUCA's COGs from Weiss et al. (2016).

**VIDEO S1.** Video of macroscopic microbial mat structures found in complete anoxic conditions at 70 m depth in the Faanu Mudugau Blue Hole (Maldives).

## Data Availability

Sequence reads data were deposited at NCBI Sequence Read Archive, with Accession Number: PRJNA1074233. Main scripts used in the bioinformatic analysis are available at https://github.com/Luponsky/Mats-Blue-Hole-Maldives/.
