# Supplementary figures and images for: Genome‐resolved metagenomics revealed novel microbial taxa with ancient metabolism from macroscopic microbial mat structures inhabiting anoxic deep reefs of a Maldivian Blue Hole
Source: Environ Microbiol Rep. 2024 Sep 12;16(5):e13315. doi: 10.1111/1758-2229.13315 (PMC11392830; doi:10.1111/1758-2229.13315)

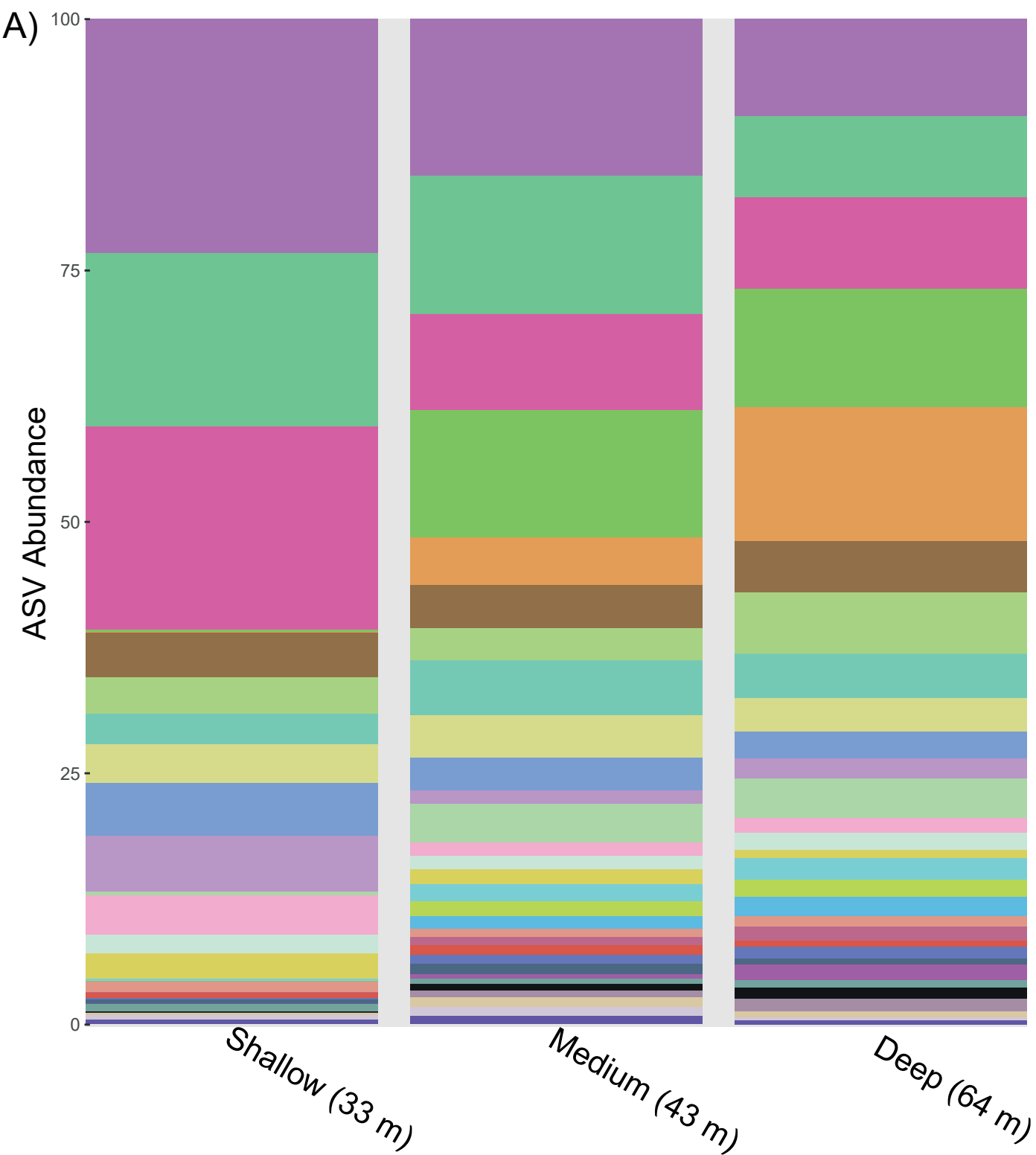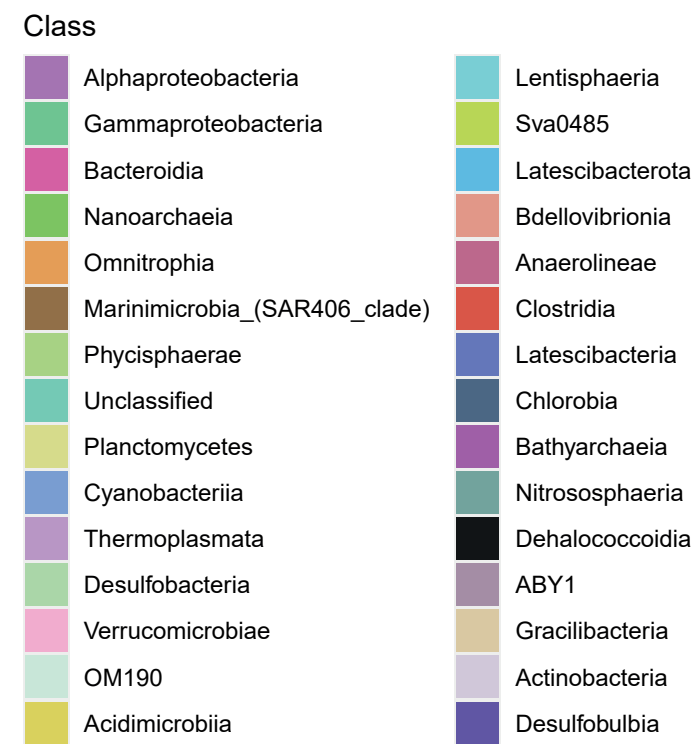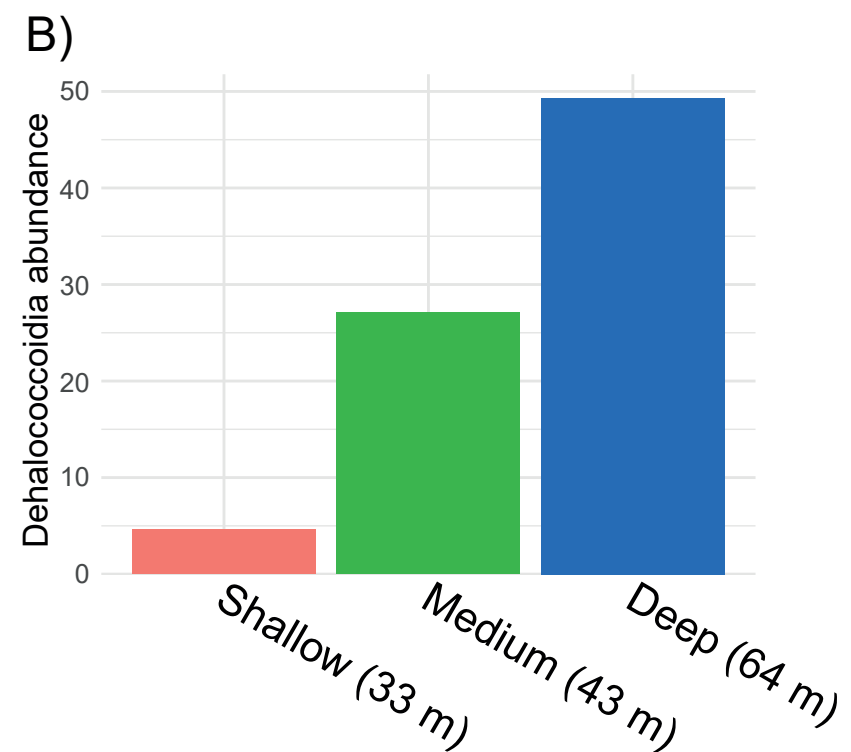

Supplement: Supplementary file 1 — FIGURE S1. Microbial community structure in the water column of the FMBH (A) Relative abundances of Bacterial ASVs in the column water sampled at depths of 33, 43 and 64 m clustered at the class level. (B) Barplot Illustrating the abundance of the Dehalococcoidia class in the column water of the FMBH. [file EMI4-16-e13315-s001.pdf]

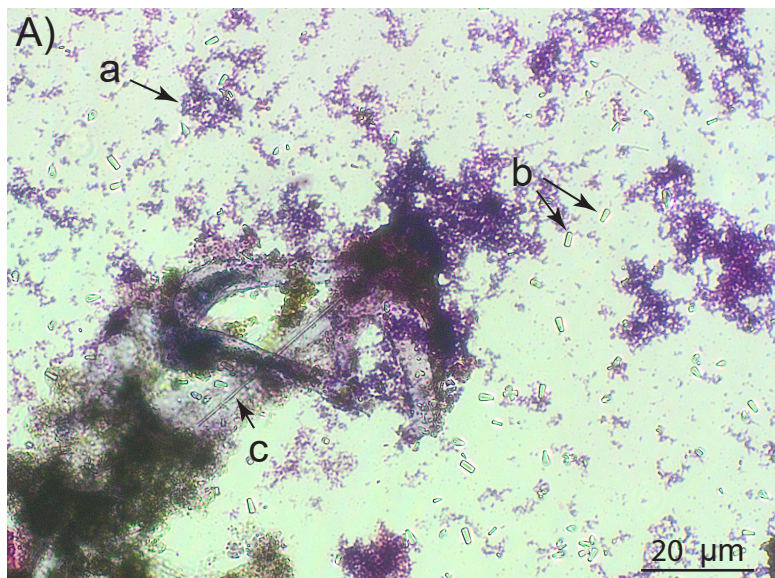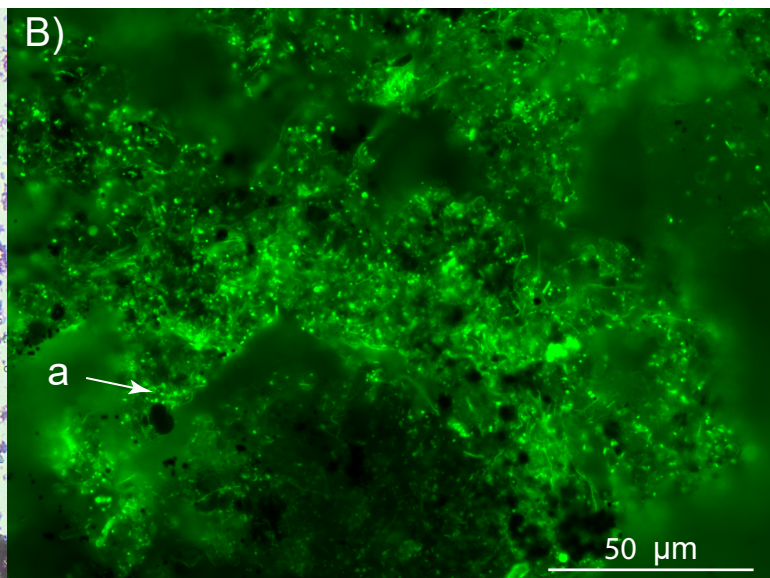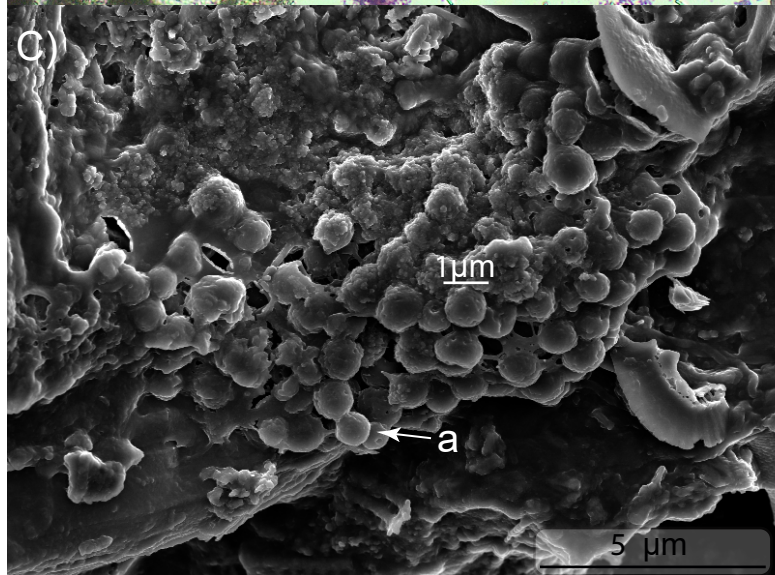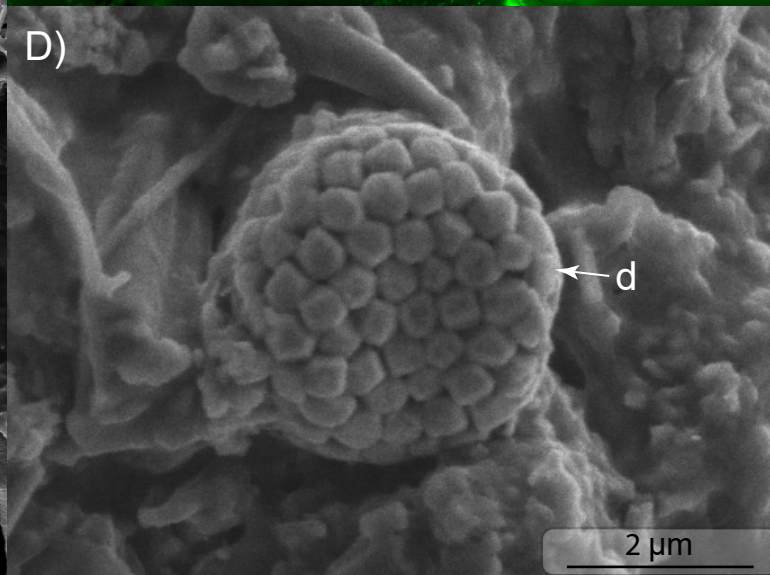

Supplement: Supplementary file 2 — FIGURE S2. Light (A, B), Epifluorescence (C) and Field emission scanning electron microscopy (FESEM) photographs of microbial mat samples collected in the FMBH. Small round‐shaped bacterial cells (~1 μm size) form a well‐discernible dense network in the mats (a), uncoloured particles (b), filamentous‐like structure (c) and nano‐micron framboidal pyrite aggregates (d) are also visible in the mat fabric. [file EMI4-16-e13315-s006.pdf]

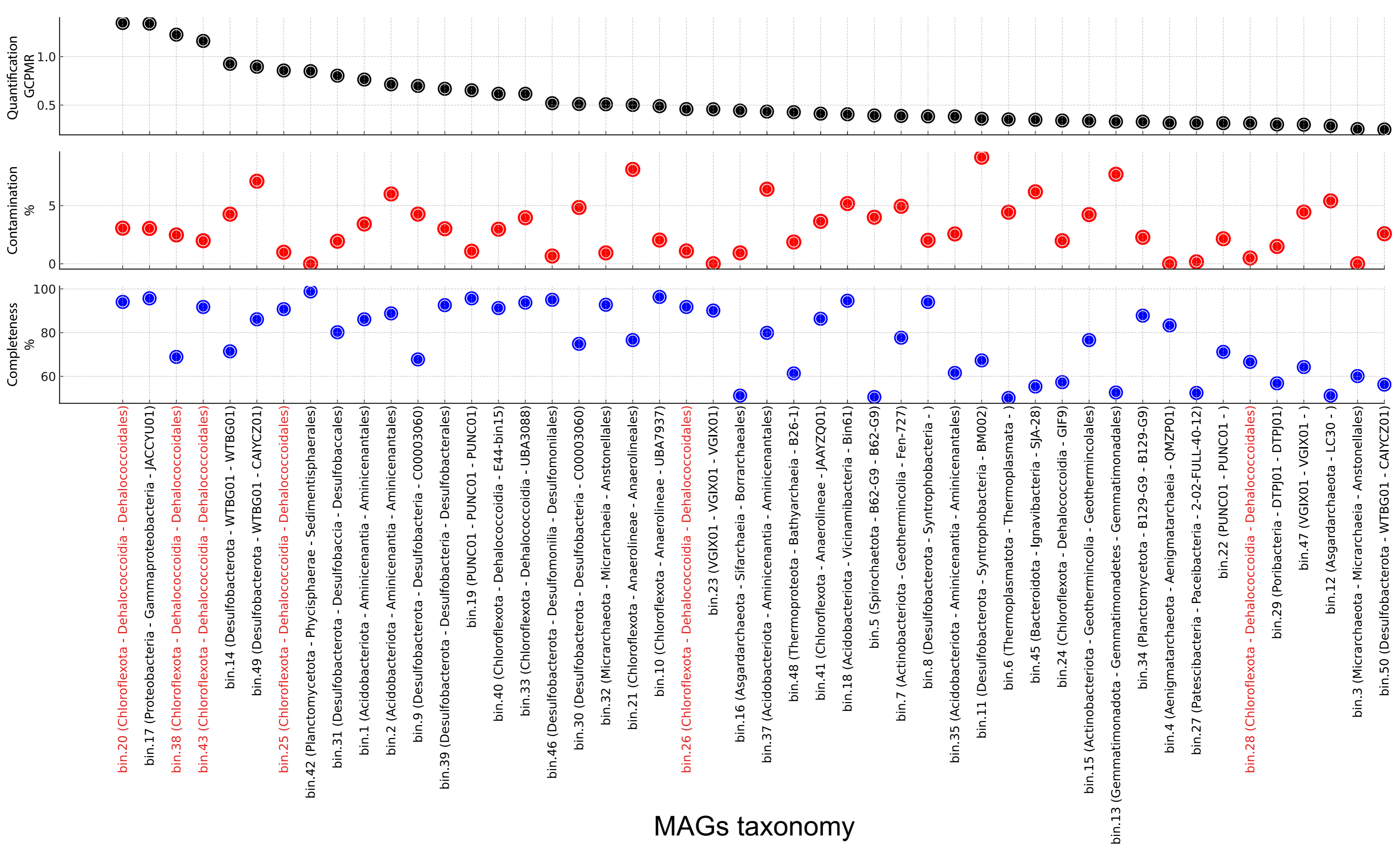

Supplement: Supplementary file 3 — FIGURE S3. MAG statistics and taxonomy. For each MAG, the figure shows quantification, contamination (%), completeness (%), and taxonomy, including phylum, class and order ranks. [file EMI4-16-e13315-s005.pdf]

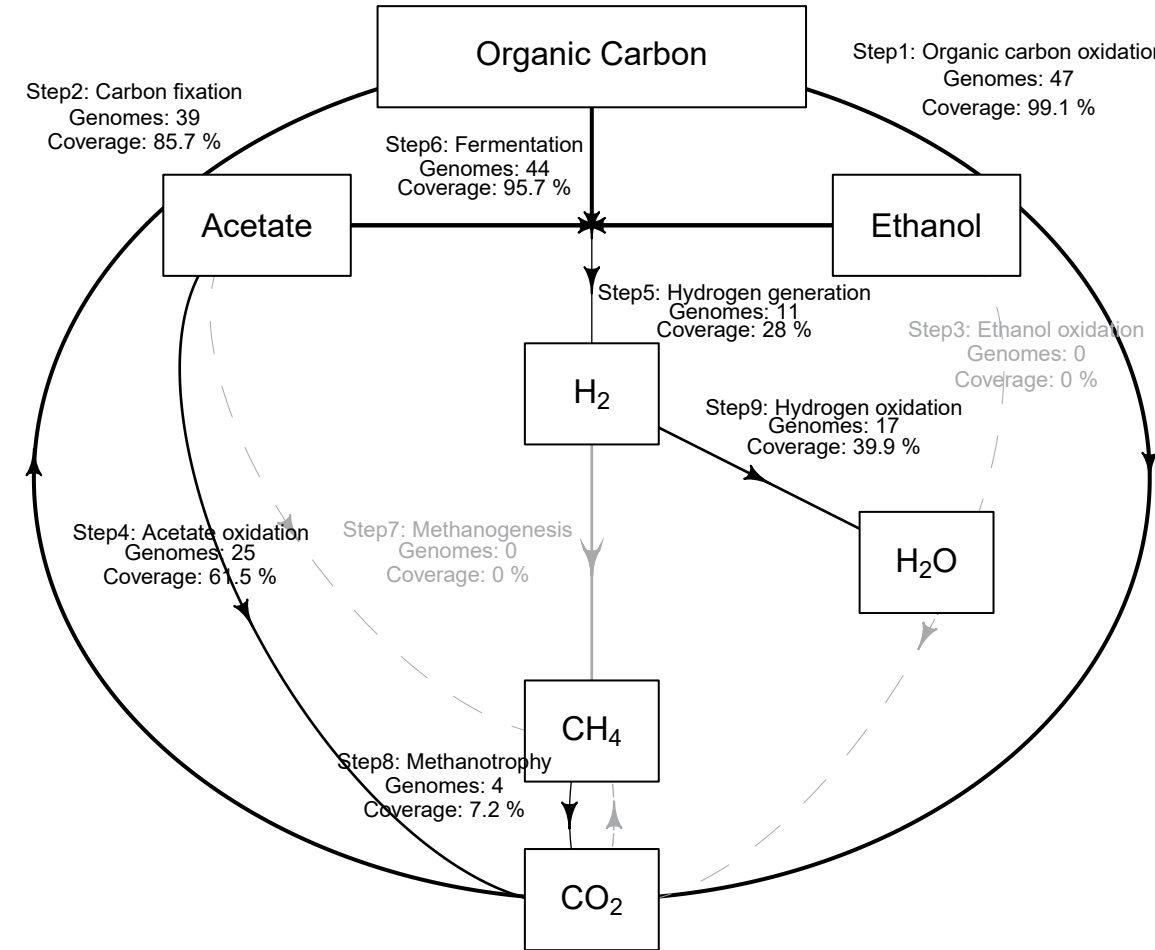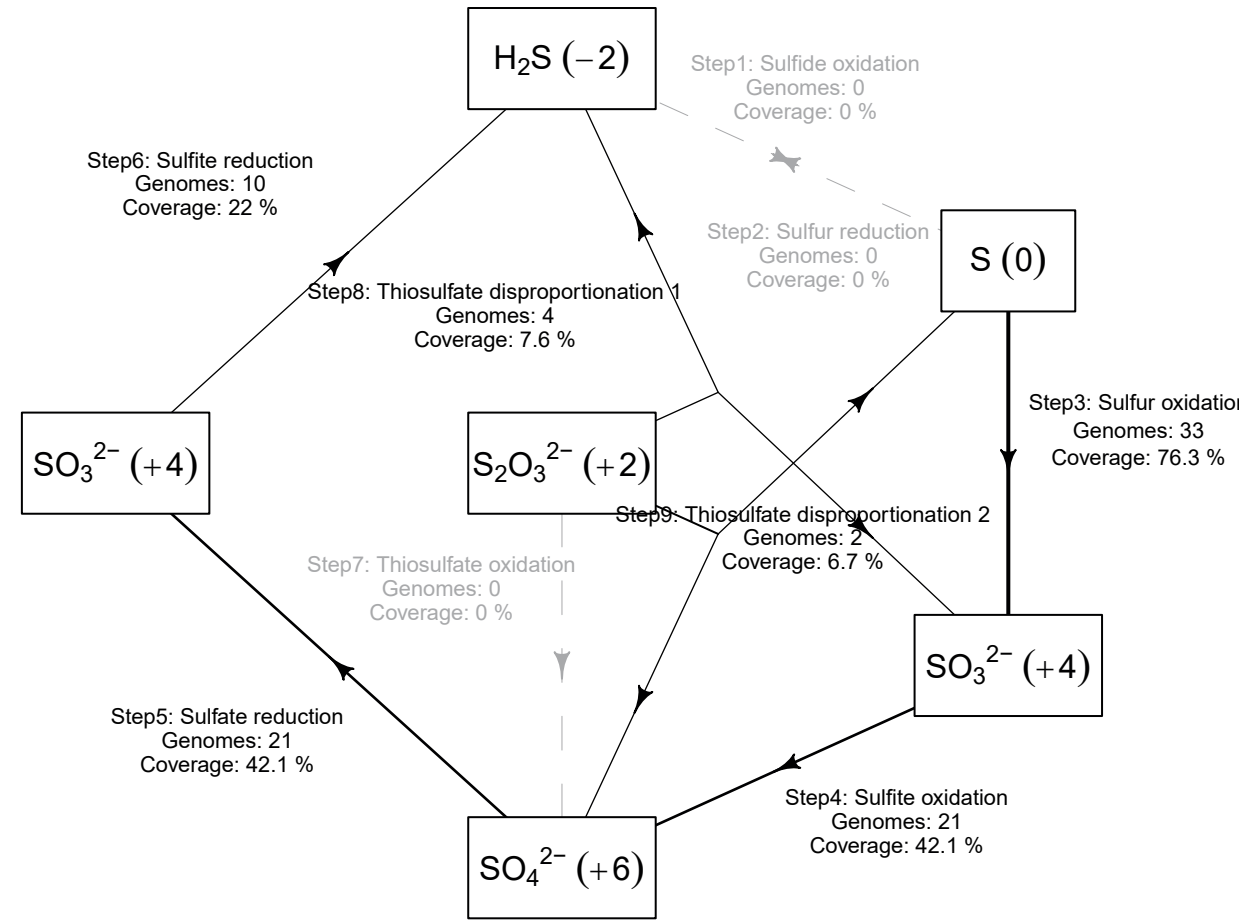

Supplement: Supplementary file 4 — FIGURE S4. Reconstruction of the carbon, sulphur, and nitrogen biogeochemical cycles at the mat community scale. [file EMI4-16-e13315-s002.pdf]
